# Epigenetic Control of Autophagy Related Genes Transcription in Pulpitis via JMJD3

**DOI:** 10.3389/fcell.2021.654958

**Published:** 2021-08-09

**Authors:** Bei Yin, Qingge Ma, Lingyi Zhao, Chenghao Song, Chenglin Wang, Fanyuan Yu, Yu Shi, Ling Ye

**Affiliations:** ^1^State Key Laboratory of Oral Diseases, West China Hospital of Stomatology, Sichuan University, Chengdu, China; ^2^West China School of Stomatology, Sichuan University, Chengdu, China

**Keywords:** pulpitis, autophagy, epigenetics, histone methylation, JMJD3

## Abstract

Autophagy is an intracellular self-cannibalization process delivering cytoplasmic components to lysosomes for digestion. Autophagy has been reported to be involved in pulpitis, but the regulation of autophagy during pulpitis progression is largely unknown. To figure out the epigenetic regulation of autophagy during pulpitis, we screened several groups of histone methyltransferases and demethylases in response to TNFα treatment. It was found JMJD3, a histone demethylase reducing di- and tri-methylation of H3K27, regulated the expression of several key autophagy genes via demethylation of H3K27me3 at the gene promoters. Our study highlighted the epigenetic regulation of autophagy genes during pulpitis, which will potentially provide a novel therapeutic strategy.

## Introduction

Autophagy is a conserved degradation/self-eating pathway delivering unwanted cytoplasmic components and organelles to lysosomes for digestion. Autophagy ensures organelle renewal and sustains the cellular homeostasis. Excessive or deficient autophagy may contribute to pathogenesis, such as cancers, inflammation, immune diseases and etc.

Autophagy is closely related to inflammation. On the one hand, several proinflammatory cytokines can induce autophagy, such as tumor necrosis factor (TNF) (Mostowy et al., 2011), interleukin1β (IL1β) (Hartman and Kornfeld, 2011) and interferons (Singh et al., 2010). On the other hand, autophagy facilitates the cell autonomous control of inflammation by removing the damaged mitochondria [thus alleviating the release of inflammasome activators such as reactive oxygen species (ROS) or mitochondrial DNA (mtDNA)] (Netea-Maier et al., 2016), degrading the aggregated inflammasomes and interferon regulatory factor 1 (IRF1) (Liang et al., 2019), etc.

Pulp is the only soft tissue in the tooth. It has four principal functions: forming dentin, providing nutrition; sensory function; defense function. Pulpitis is the inflammation of the dental pulp caused by deep caries, trauma, dental fissures, etc. It is one of the most common dental disorders and usually causes severe pain. Generally, the current treatment of pulpitis is root canal therapy in which the dental pulp is cleared away. Loss of the vital pulp may result in postoperative pain, root fracture, secondary infection, leading to a higher incidence of the tooth extraction (Nakashima et al., 2017). In order to preserve the pulp vitality, researchers have focused on studying the underlying regulation mechanisms of the pulpitis pathogenesis. Recently, it was found autophagy was increased during the inflammation process of dental pulp. Autophagy related genes such as autophagy related 5 (ATG5), ATG7, microtubule associated protein 1 light chain 3 (LC3) and beclin 1 (BECN1) were increased in pulpitis tissue (Qi et al., 2019). The role of autophagy in pulpitis may be dual. Autophagy was induced in odontoblast at the early stage (6 h treatment) of lipopolysaccharide (LPS) stimulation. Autophagy of this stage acted as a protector to conserve cell viability. On the contrary, autophagy was down-regulated in the late-stage (12 h treatment) of LPS treatment, when autopahgy promoted cell death (Pei et al., 2015). Therefore, autophagy may possibly be fine tuned by certain mechanisms to maintain the homeostasis of pulp tissue.

The regulation of autophagy in pulpitis has been studied by several reports. Both the transcription factor forkhead box O3 (FOXO3) and a surface marker CD47 (a “marker of self” distinguishing host cells from foreign invaders) were reported to regulate autophagy (Wang H. et al., 2016; Li et al., 2018). Although epigenetics is one of the most important mechanism linking the extra-cellular signals to the transcription of genes, the epigenetic regulation of autophagy during pulpitis is largely unknown. Histone methylation is an important epigenetic modification for determining the chromatin accessibility and the ensuing transcriptional status. Histone methylation has important effects on the modulation of autophagy induction. Previous studies found histone methylations such as trimethylation of lysine 27 on histone 3 (H3K27me3), trimethylation of lysine 9 on histone 3 (H3K9me3) can affect the transcription of autophagy related genes (de Narvajas et al., 2013; Park et al., 2016). However, it is unknown whether autophagy in pulpitis is regulated by certain histone methylation. To figure out the epigenetic regulation of autophagy during pulpitis, we screened several groups of histone methyltransferases and demethylases in response to TNFα treatment. TNFα is an inflammatory cytokine and TNFα stimulation of HDPCS is often used as an effort to replicate the cell status of pulpitis *in vitro* (Yin et al., 2017). Several studies have reported the suppression of autophagy by TNFα (Dash et al., 2018). In contrast, there are studies reporting the induction of autophagy by TNFα (Chen D. et al., 2016). The effect was TNF on autophagy in HDPCs was unknown, while it was reported that LPS stimulation induced autopahgy in odontoblast. It was found jumonji domain containing 3 (JMJD3), a histone demethylase reducing di- and tri-methylation of H3K27, could regulate the expression of several key autophagy genes by mediating the H3K27 methylation. Our study highlighted the epigenetic regulation of autophagy genes during pulpitis, potentially providing the important clues of therapeutic targets.

## Materials and Methods

### Establishment of Rat Pulpitis Model

The study was approved by the ethics committee of the West China School of Stomatology, Sichuan University. The rat pulpitis model was established as previously (Yin et al., 2017). The pulp chamber of the first molars was opened, so the first molars acted as pulpitis group and the adjacent normal molars acted as healthy control group. The samples were fixed with 4% paraformaldehyde at 4°C for 12 h followed by dehydration, paraffin embedding and slicing.

### Immunofluorescent Staining

The samples were dewaxed with xylene, hydrated with graded ethanol, and rinsed with distilled water. Then the sections were subjected to antigen retrieval by pepsin solution at 37°C for 30 min. After treatment with 30% H2O2 and goat serum, the samples were incubated with the primary antibody overnight at 4°C. The sections were successively subjected to a fluorescent secondary antibody and 4′,6-diamidino-2-phenylindole (DAPI). Then the slides were mounted and observed under a Nikon Eclipse300 fluorescence microscope (Compix Inc, Sewickley, PA, United States).

### Cell Culture and TNFα Treatment

Primary human dental pulp cells (HDPCs) were cultured and passaged according to our previous study (Yin et al., 2017). The third and fourth passage of the cells were used in our study. For TNFα stimulation, cells were treated with human recombinant tumor necrosis factor α (TNFα) (10 ng/mL) (R&D, Minneapolis, MN, United States) in the presence of GSKJ-4 (Sigma-Aldrich, MO, United States) or DMSO in a serum-free medium for 2 h unless indicated.

### Quantitative Real-Time Polymerase Chain Reaction (qRT-PCR)

The total RNA was extracted using the RNeasy mini kit (Qiagen, Valencia, CA, United States). After assessing the concentration and purity of RNA, cDNA was synthesized using the HiScript III SuperMix (Vazyme Biotech, Nanjing, China). Real-time polymerase chain reaction was done using ChamQ Universal SYBR qPCR Master Mix (Vazyme). Conditions for qRT-PCR were as follows: denaturation at 95°C for 30 s, 40 cycles at 95°C 10s and 60°C 30 s. The relative expression level of mRNA is presented as the fold change of the target gene relative to the control calculated by the formula x = 2^–^^Δ^ ^Δ^ ^Ct^ after glyceraldehyde-3-phosphate dehydrogenase (GAPDH) correction.

### Western Blot

The protein was extracted using the Mammalian Protein Extraction Reagent (Thermo Fisher Scientific, Hudson, NH, United States). The loading volume was calculated (20 mg/lane) based on the protein concentration which was determined by a BCA Assay Kit (Beyotime Biotechnology, Beijing, China). The samples were electrophoresed and then transferred to a polyvinylidene fluoride membrane (Millipore, Billerica, MA, United States). The membranes were immersed in 5% BSA for 1 h of blocking and incubated with primary antibodies for JMJD3, FIP200, BECLIN, ATG5, H3K27me3, H3 and GAPDH (all 1:1000) (all from Cell Signaling Technology, Danvers, MA, United States). The incubation took place at 4°C overnight. Membranes were washed and incubated with appropriate HRP-conjugated immunoglobulin G antibodies (Abcam) before visualizing with High Sensitive ECL Chemiluminescent Substrate (Vazyme).

### Infection of Ad-GFP-LC3B

HDPCs of 70% confluence were infected with an adenovirus expressing GFP-LC3B (Ad-GFP-LC3B) (Beyotime). After 24 h of infection, HDPCs underwent the corresponding treatments such as TNFα, GSKJ-4, etc. Then the nuclei were stained with DAPI, photographed by a fluorescence microscope and quantified with Image J.

### Small Interfering RNA (siRNA) Transfection

Cells at 90% confluence were transfected according to the manuals of Lipofectamine 3000. JMJD3 stealth siRNA (HSS177200) and negative siRNA (12935200, all from Thermo Fisher) were used in our study. 6 h after transfection, the cells were treated with 10 ng/mL TNFα or phosphatebuffered saline (control) for 2 h before the extraction of RNA and protein.

### Chromatin Immunoprecipitation (ChIP)

ChIP experiments were performed using the Magna ChIP^TM^ HiSens (Millipore) based on its protocols. Briefly, the cells were cross-linked with 37% paraformaldehyde and then the cell pellets were lysed with EZ-Zyme^TM^ Lysis Buffer. After that, the EZ-Zyme^TM^ Digestion Buffer containing EZ-Zyme^TM^ Enzymatic Cocktail was used for nuclease digestion. Precipitation reaction was performed at 4°C overnight containing 490 μL SCW buffer, 10 μl resuspended A/G Magnetic Beads, 5 μl antibody (JMJD3, H3K27me3, rabbit IgG) and 5 μl digested chromatin. Then the samples were subjected to de-crosslinking using Proteinase K. The supernatant was collected for qRT-PCR using primers that targeted the promoters of the following genes. The ChIP-qPCR primer sequence was as follows: ATG5: F:5′-AGGCAATGCACCTTAATCCCAC-3′, R:5′-GC AGAAATCCTCACTACAGTGTC-3′; LC3B: F:5′-CTGTAAA CCACCCACCACCA-3′, R:5′-CTGAAGTGTGTGTGTGCTGC-3′; FIP200: F:5′-GGTATGAACCAGTCGTTTCTGG-3′, R:5′ -TCTGAACTATGCCAGTGATAATCT-3′; ATG12: F:5′-CCCA TTCGGGAGGATCAACT-3′, R:5′-TTCTGCTACTCGTGTG TGGT-3′; ATG7: F:5′-GTCCAGGCTGTTCTTGGTCA-3′, R:5′ -CCCCTGAATGCCCATTCCTC-3′; BECLIN: F:5′-AGTTATG TGCAAGCACTTTGGAA-3′, R:5′-TGCAATGAAGAGCTGGC TAC-3′.

### Statistical Analysis

The SPSS software was used for statistical analysis, and one-way analysis of variance test was done in our study. Statistical difference (*p* < 0.05) and significant statistical difference (*p* < 0.01) were represented as ^∗^ and ^∗∗^ respectively. Data were presented as the mean ± standard deviation.

## Results

### Expression of LC3B and Autophagy-Related Genes in Response to TNFα Stimulation

**FIGURE 1:**
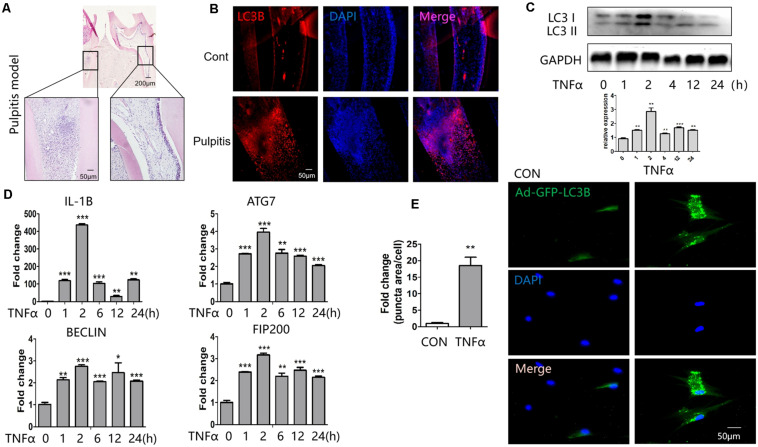
Expression of LC3B and autophagy-related genes in response to TNFα stimulation. **(A)** Hematoxylin-eosin stained sections of healthy (right) and inflamed (left) dental pulp. **(B)** Immunofluorescent staining revealed enhanced expression of LC3B in pulpitis tissue (down) compared with control pulp (up). **(C)** Western blot analysis of LC3B expression at indicated time point of TNFα stimulation in HDPCs. Below was the quantification of the bands relative to the control. **(D)** QPCR analysis of inflammatory and autophagic genes at indicated time point of TNFα stimulation in HDPCs. **(E)** GFP-LC3 puncta was enhanced in response to TNFα stimulation in GFP-LC3B-expressing HDPCs. The relative quantification was on the left side. *P < 0.05, **P < 0.01, ***P < 0.001.

To determine the alteration of autophagy during pulpitis, we established the rat pulpitis model. Hematoxylin-eosin staining was done to verify the infection, that is the infiltration of inflammatory cells (Figure 1A). Immunofluoresent staining revealed that LC3B expression was enhanced in pulpitis tissue compared with the adjacent healthy control (Figure 1B).

To study the regulation of autophagy *in vitro*, we cultured HDPCs and stimulated them with inflammatory cytokine TNFα. It turned out that the expression of LC3B peaked at 2 h after stimulation of TNFα (Figure 1C). The qPCR results showed that the proinflammatory gene IL1β was highly induced after TNFα stimulation for 2 h (Figure 1D). Meanwhile, the expression of the autophagy related genes such as autophagy related 7 (ATG7), BECLIN 1, 200-kDa FAK-family interacting protein (FIP200) was enhanced after TNFα treatment (Figure 1D). Notedly, the expression of these genes also peaked at 2 h of TNFα stimulation. To observe autophagy vesicles in response to TNFα stimulation, we transfected HDPCs with Ad-GFP-LC3B. GFP-tagged LC3B reporters are widely used for the measure of autophagy. Upon autophagy induction, the cytosolic GFP-LC3-I is conjugated to phosphatidylethanolamine (PE) and thus converted to LC3-II. LC3-II then tethers to the membranes of autophagosomes and thus presents fluorescent puncta signal (Adiseshaiah et al., 2018). Measuring the fluorescent puncta can therefore reflects the autophagosomes. Through this measurement, we found TNFα treatment significantly increased autophagy vesicles (Figure 1E).

### JMJD3 Expression Was Prominently Induced in Response to TNFα Stimulation

**FIGURE 2:**
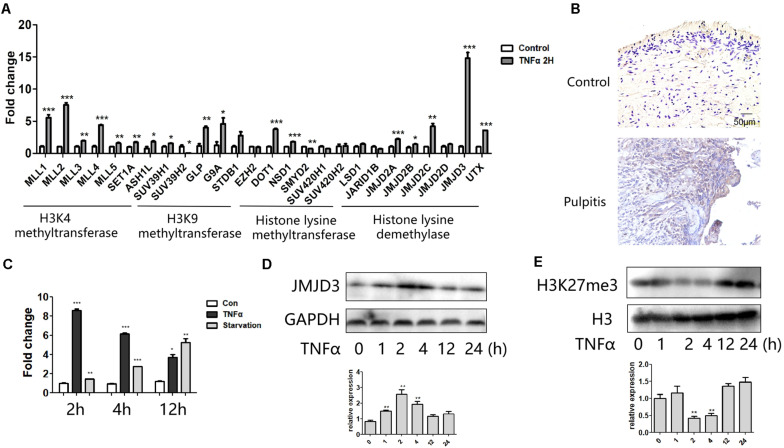
JMJD3 expression was most highly induced in response to TNFα stimulation. **(A)** QPCR analysis of several groups of histone lysine methyltransferase and demethylases in HDPCs with 2 h stimulation of TNFα. **(B)** Immumohistochemical staining of JMJD3 in healthy control pulp tissue (up) and pulpitis tissue (low). **(C)** QPCR analysis of JMJD3 expression in HDPCs with TNFα or starvation stimulation at indicated time. **(D,E)** Western blot analysis of JMJD3 **(D)** and H3K27me3 **(E)** in HDPCs with TNFα stimulation. Below was the relative quantification of the bands. ^∗∗^*P* < 0.01, ^∗∗∗^*P* < 0.001.

We profiled the expression of several groups of histone lysine methyltransferases in response to TNFα. We found a pronounced level of jumonji domain containing 3 (JMJD3), an H3K27 demethylase, was obviously elicited among these epigenetic regulators (Figure 2A). Immunohistochemical staining showed that JMJD3 expression was augmented in human pulpitis tissue (Figure 2B). Interestingly, JMJD3 expression also peaked at 2 h stimulation of TNFα treatment (Figures 2C,D). And starvation of the HDPCs as a positive control also induced the expression of JMJD3. Conversely, the substrate of JMJD3, H3K27me3, was markedly decreased at 2 h stimulation of TNFα (Figure 2E). We noted that the level of H3K27me3 was not always reversely correlated with the level of JMJD3 at various time point. Possibly, the level of H3K27me3 in response to TNFα treatment was affected by both H3K27 methylase EZH2 and H3K27 demethylases, JMJD3 and UTX. EZH2 was reported to increase upon TNFα stimulation in human dental pulp cells (Hui et al., 2014). UTX was also reported to play crucial roles in TNFα signaling in endothelial cells (ECs) (Higashijima et al., 2020). Therefore, the level of H3K27me3 in response to TNFα stimulation in HDPCs may occur as the result of the coordination of those H3K27 methylases and demethylases.

Since H3K27me3 modification was reported to occur in the promoter regions of several autophagy related genes, we wondered whether JMJD3 played a role in regulating autophagy related gene expression in HDPCs.

### GSKJ-4 Mediated the Decrease of Autophagy Genes

**FIGURE 3:**
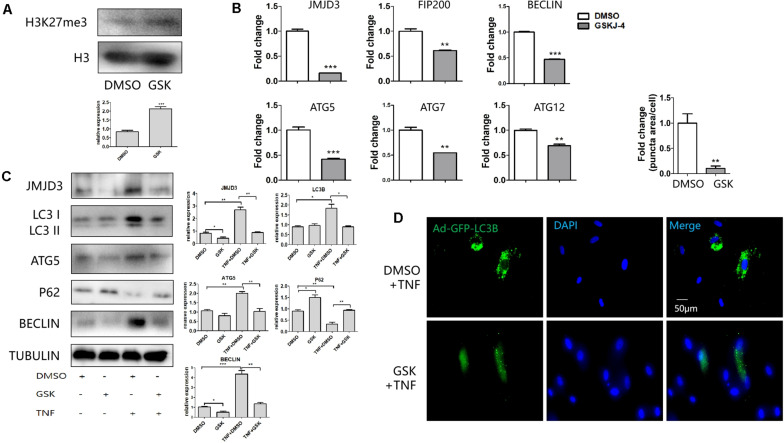
GSKJ-4 mediated the decrease of autophagy genes. **(A)** Western blot analysis of H3K27me3 level in response to GSKJ-4 treatment. Below was the relative quantification of the bands. **(B,C)** QPCR **(B)** and western blot **(C)** analysis of JMJD3 and several autophagic genes after the treatment of GSKJ-4. The relative quantification was on the right. **(D)** GFP-LC3 puncta and the corresponding quantitative analysis (up) in response to GSKJ-4 treatment. The relative quantification was on the upper side. **P < 0.01, ***P < 0.001.

GSKJ-4 was the inhibitor of JMJD3. As expected, treatment of GSKJ-4 caused the increase of H3K27me3 (Figure 3A). The expression of FIP200, BECLIN, ATG5, ATG7, ATG12 was decreased in mRNA level in response to GSKJ-4 treatment (Figure 3B). The western blot revealed that the expression of FIP200, BECLIN, ATG5, LC3B and JMJD3 was enhanced in TNFα group. GSK J-4 treatment led to the decreased expression of JMJD3, ATG5, LC3B, BECLIN and JMJD3 (Figure 3C). The decrease of the genes in response to GSKJ-4 was more obvious in the group of TNFα treatment than the group without TNFα stimulation. The P62 is an autophagic adapter sequestering polyubiquitinated proteins and binds directly to LC3. Therefore, P62 acts as an autophagy-specific substrate. Western blot showed that TNFα stimulation led to the decrease of P62, while JMJD3 inhibition resulted in the increase of P62. AdGFP-LC3B infection also revealed that after GSKJ-4 treatment the autophagy vesicles were diminished (Figure 3D). Taken together, the results indicated that the enzymatic activity of JMJD3 regulated the autophagy gene expression during pulpitis by mediating the expression of autophagy genes.

### JMJD3 siRNA Regulated the Decrease of Autophagy Genes

**FIGURE 4:**
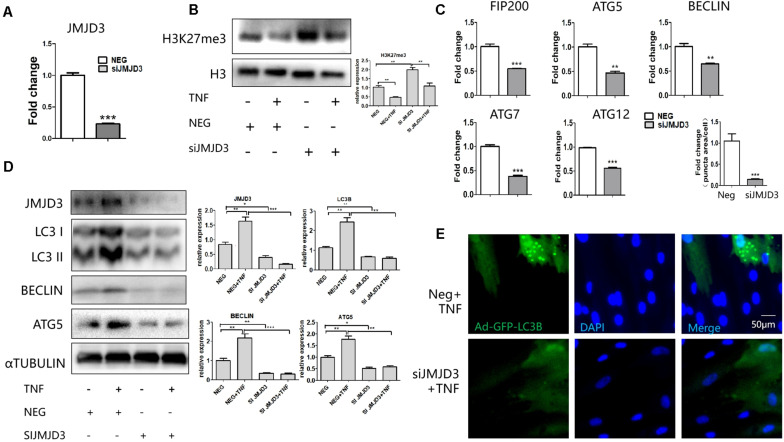
JMJD3 siRNA regulated the decrease of autophagy related genes. **(A)** QPCR analysis of JMJD3 level after JMJD3 siRNA transfection. **(B)** Western blot analysis of H3K27me3 level after the treatment of JMJD3 siRNA. The relative quantification was on the right. **(C,D)** QPCR **(C)** and western blot **(D)** analysis of autophagy related genes in response to JMJD3 siRNA. The relative quantification was on the right. **(E)** GFP-LC3B-expressing HDPCs cells were treated with JMJD3 siRNA for 24 h followed by TNFα or PBS treatment. The relative quantification was on the upper side. **P < 0.01, ***P < 0.001.

JMJD3 siRNA was transfected to evaluate whether knockdown of JMJD3 would affect the autophagy process. The expression of JMJD3 was depleted in the siRNA-treated group (Figure 4A). The alteration of H3K27me3 level coincided with JMJD3’s role as an H3K27 demethylase (Figure 4B). Consistent with the GSKJ-4 treatment, JMJD3 suppression decreased the expression of FIP200, BECLIN, ATG5, ATG7, ATG12 with or without TNFα treatment (Figures 4C,D). Meanwhile, the LC3 puncta was also decreased in the JMJD3 siRNA-treated group (Figure 4E). Furthermore, we used the LPS from P. gingivalis to study the role of JMJD3 in pulpitis. Consistent with TNFα treatment, LPS stimulation also up-regulated the expression FIP200, BECLIN, ATG5 (Supplementary Figure 1A). Silencing of JMJD3 decreased their expression in both the protein and RNA level (Supplementary Figures 1A,B). Taken together, JMJD3 knockdown regulated the decrease of autophagy genes.

### JMJD3 Regulated the Expression of Autophagy-Related Genes by Regulating the H3K27me3 Modification

**FIGURE 5:**
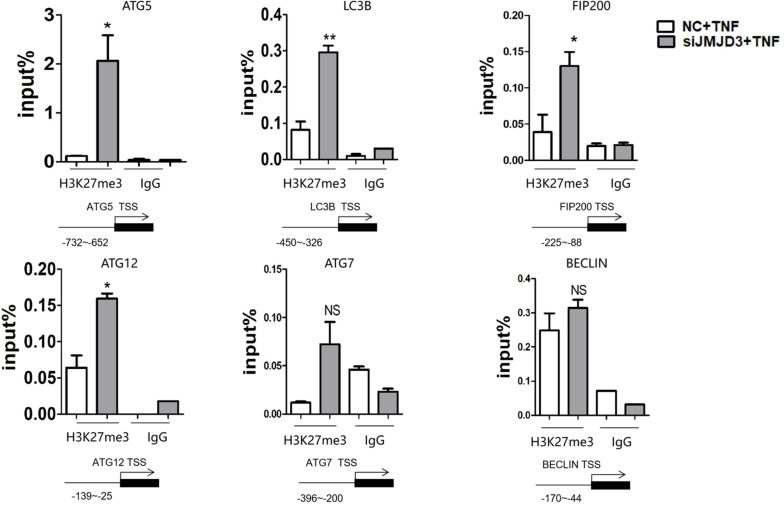
JMJD3 regulated the expression of autophagy-related genes by regulating the H3K27me3 modification. JMJD3 siRNA up-regulated the H3K27me3 level binding to the promoters of ATG5, FIP200, ATG12 and LC3B. *P < 0.05, **P < 0.01, ns non-significant.

To identify whether JMJD3 could regulate the expression of autophagy-related genes directly, we performed ChIP analysis. It turned out that the silencing of JMJD3 upregulated the H3K27me3 level at the promoter region of ATG5, LC3B, FIP200 and ATG12. Although the H3K27me3 modification was present at the BECLIN promoter, JMJD3 silencing didn’t alter the H3K27me3 enrichment (Figure 5). The H3K27me3 modification on BECLIN gene may be possibly mediated by other H3K27demethylases such as lysine demethylase 6A (KDM6A). As for ATG7 gene, we didn’t detect any H3K27me3 modifications at the promoter regions. These findings suggested that JMJD3 could regulate the H3K27me3 modification on ATG5, LC3B, FIP200 and ATG12.

## Discussion

Autophagy plays an important role in maintaining cellular homeostasis in teeth. Besides the above-mentioned pulpitis, autophagy is reported to participate in tooth development (Yang et al.,2013a,b) and aging (Murray et al., 2002; Couve and Schmachtenberg, 2011; Couve et al., 2012). Therefore, figuring out the autophagy regulators are of great significance to the teeth homeostasis. Rapamycin is a well-recognized autophagy inducer with great potential for various diseases, but the side effects of hyperglycemia, hyperlipidemia, insulin resistance, etc. motivated the researchers to find alternative therapeutic autophagy inducer (Salmon, 2015). Our study revealed autophagy may be dynamically regulated in pulpitis by using small molecule compound targeting the epigenetic regulators, potentially highlighting a novel therapeutic strategy in the treatment of pulpitis. Our study found GSKJ-4, the small molecule inhibitor of JMJD3, not only changed the H3K27me3 level but decreased the level of JMJD3 as well. Consistent with our results, GSKJ-4 treatment did decrease the expression of JMJD3 in fibroblast-like synoviocytes (Jia et al., 2018), breast cancer stem cells (Yan et al., 2017) and renal interstitial fibroblasts *in vitro* (Yu et al., 2021). Furthermore, GSKJ4 administration by intraperitoneal injection down-regulated JMJD3 level in the kidney *in vivo*. However, in several cells such as endothelial progenitor cells (He et al., 2020) and hepatocytes (Pediconi et al., 2019), GSKJ4 treatment has no impact on the expression of JMJD3. This discrepancy is possibly due to the cell type-dependent responses of transcription factors (TFs) in response to GSK-J4 treatment. GSK-J4 treatment can affect the expression of several TFs including signal transducer and activator of transcription (STAT) (Das et al., 2017). STAT can bind to the JMJD3 promoter and regulates the transcription of JMJD3 (Przanowski et al., 2014; Sherry-Lynes et al., 2017). Therefore, the downregulation of JMJD3 in response to GSK-J4 treatment may be attributed to the GSK-J4-mediated TFs such as STAT.

Our study found TNFα treatment stimulated autophagy in HDPCs, consistent with Serge’s study in TNFα-stimulated HeLa cells (Mostowy et al., 2011). We found LC3B puncta were significantly increased in pulpitis tissue compared with the healthy control. Consistently, Wang found LC3 expression could only be detected in caries and pulpitis groups rather than in healthy samples. Possibly, autophagy may be quiescent or less active under normal physiological conditions. Once the external stimuli interrupted the tissue homeostasis, autophagy may be activated elaborately by complicated mechanisms including epigenetics. Mechanismly, we found JMJD3 could regulate the key autophagy genes by decreasing the H3K27me3 modification at the promoters of ATG5, LC3B, FIP200 and ATG12. Consistent with this, Denton found another H3K27 demethylase, KDM6A, was recruited to the promoters of autophagy genes such as ATG5 and LC3B, regulating the enrichment of H3K27me3 on their promoters (Denton et al., 2013). Similarly, the H3K36 demethylase lysine demethylase 4A (KDM4A) was found to repress expression of LC3B and BECLIN1 by H3K36 demethylation (Wang B. et al., 2016). An H3K9 methyltransferase euchromatic histone lysine methyltransferase 2 (EHMT2) also suppressed BECLIN-1 expression by reducing H3K9me2. In addition, other modifications such as H3K4me3, H3K27 acetylation (H3K27ac), H4K16ac and H3K56ac were also correlated with the mRNA expression of several autophagy genes (Füllgrabe et al., 2013; Peeters et al., 2019). Interestingly, several autophagy genes including LC3B possess bivalent modification with high H3K4me3 and H3K9me3 levels. The bivalent modification can poise the chromatin conformation for immediate response to stimuli (Biga et al., 2017). Therefore, the autophagy genes can be dynamically adjusted to the changing environment in the transcription level. Interestingly, it was found JMJD3 interference affected the expression of ATG7 and BECLIN without regulating the H3K27me3 level around their promoters. Possibly, JMJD3 may regulate their expression by targeting the upstream modulators of autophagy. For example, enhancer of zeste 2 polycomb repressive complex 2 subunit (EZH2) can affect the autophagy genes by tuberous sclerosis 2 (TSC2)/mammalian target of rapamyoin (MTOR) pathway (Wei et al., 2015). H3K4 demethylase lysine-specific demethylase 1 (LSD1) can bind to the promoter region of Sestrin2 (SESN2) and regulate autophagy through SESN2/MTOR pathway (Ambrosio et al., 2017).

Apart from mediating histone demethylation, JMJD3 was also reported to regulate non-histone proteins such as the retinoblastoma (RB) protein at the lysine810 residue (K810) (Zhao et al., 2015). Besides, JMJD3 can directly interact with p53 and induce p53 stabilization (Sola et al., 2011). The autophagy components can be modified to dictate the autophagic cascade (Morselli et al., 2011; Lin et al., 2012). For example, ATG3 protein could be acetylated in lysine 19 (K19) and K48, thus affecting the ATG3 and ATG8 interaction (Yi et al., 2012). Several other autophagy components such as ATG5, ATG, ATG8, and ATG12 can also be acetylated (Lee et al., 2008; Lee and Finkel, 2009). It will be intriguing to figure out whether JMJD3 can demethylase autophagy-related proteins directly.

It’s worth noting JMJD3 mediation of the autophagy-related genes may potentially have some non-autophagy function. For example, Gan found FIP200 protected cells from the TNFα-induced apoptosis in an autophagy independent way (Chen S. et al., 2016; Cadwell and Debnath, 2018). Another study found ATG5 decreased the amount of neutrophils during Mycobacterium tuberculosis infection independent of autophagy function (Kimmey et al., 2015). Therefore, further studies are needed to clarify whether ATG5 and FIP200 regulated by JMJD3 may possibly exert the above-mentioned non-autophagic roles in pulpitis.

It would be interesting to study whether combined intervention JMJD3 and other epigenetic factors may present a better way to control autophagy. In fact, it was reported that the inhibitors of histone H3K4 demethylase LSD1 could induce autophagy in multiple mammalian cell lines (Wang et al., 2017). Likely, JMJD3 could coordinate with H3K4 methyltransferases to promote the transcription of autophagy genes in the TNFα-stimulated HDPCs. This coordination may be accomplished by JMJD3 recruiting the Set1/MLL H3K4 methyltransferase complexes (Shi et al., 2014) and incorporating in the MLL complex (De Santa et al., 2007). Taken together, our study highlighted the epigenetic regulation of autophagy genes during pulpitis, potentially providing the important clues of therapeutic targets.

## Data Availability Statement

The original contributions presented in the study are included in the article/Supplementary Material, further inquiries can be directed to the corresponding author/s.

## Ethics Statement

The animal study was reviewed and approved by The Ethics Committee of the West China School of Stomatology, Sichuan University.

## Author Contributions

BY: conception and design, collection and assembly of data, and manuscript writing. LZ, QM, CS, and FY: data analysis and interpretation. CW, YS, and LY: final approval of manuscript. All authors contributed to the article and approved the submitted version.

## Conflict of Interest

The authors declare that the research was conducted in the absence of any commercial or financial relationships that could be construed as a potential conflict of interest.

## Publisher’s Note

All claims expressed in this article are solely those of the authors and do not necessarily represent those of their affiliated organizations, or those of the publisher, the editors and the reviewers. Any product that may be evaluated in this article, or claim that may be made by its manufacturer, is not guaranteed or endorsed by the publisher.
