# Supplementary material for: Epigenetic Control of Autophagy Related Genes Transcription in Pulpitis via JMJD3
Source: Front Cell Dev Biol. 2021 Aug 9;9:654958. doi: 10.3389/fcell.2021.654958 (PMC8381646; doi:10.3389/fcell.2021.654958)
Supplement: Supplementary file 1 [file Data_Sheet_1.PDF]

Supplemental data

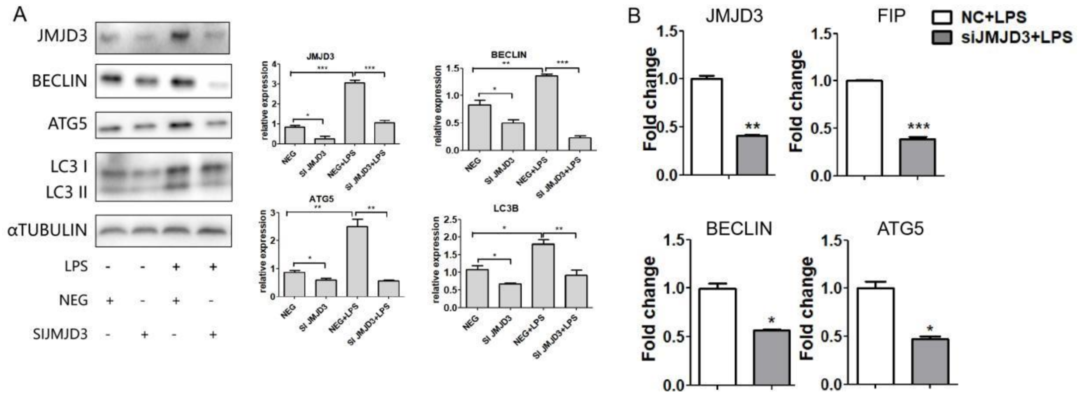


WB (A) and QPCR (B) analysis of autophagy related genes in response to JMJD3 siRNA with or without LPS treatment.
